# Predictive value of resting-state fMRI graph measures in hypoxic encephalopathy after cardiac arrest

**DOI:** 10.1016/j.nicl.2025.103763

**Published:** 2025-03-05

**Authors:** Puck Lange, Marlous Verhulst, Anil Man Tuladhar, Prejaas Tewarie, Hanneke Keijzer, Catharina J.M. Klijn, Cornelia Hoedemaekers, Michiel Blans, Bart Tonino, Frederick J.A. Meijer, Rick C. Helmich, Jeannette Hofmeijer

**Affiliations:** a Department of Clinical Neurophysiology, University of Twente, Faculty of Science and Technology, 7522 NB Enschede, the Netherlands; b Department of Neurology, Donders Institute for Brain, Cognition, and Behaviour, Radboud University Medical Centre, 6500 HC Nijmegen, the Netherlands; c Department of Neurology, Rijnstate Hospital, 6800 TA Arnhem, the Netherlands; d Department of Intensive Care Medicine, Radboud University Medical Centre, 6500 HC Nijmegen, the Netherlands; e Department of Intensive Care Medicine, Rijnstate Hospital, 6800 TA Arnhem, the Netherlands; f Department of Radiology, Rijnstate Hospital, 6800 TA Arnhem, the Netherlands; g Department of Medical Imaging, Radboud University Medical Centre, 6500 HC Nijmegen, the Netherlands

**Keywords:** Postanoxic coma, Cardiac arrest, Functional MRI, Functional connectivity, Graph theory

## Abstract

**Introduction:**

Current multimodal prediction models can determine the prognosis of about half of comatose cardiac arrest patients. We investigated whether whole-brain graph-theoretical measures from early resting-state functional magnetic resonance imaging (fMRI) three days after cardiac arrest discriminate between good and poor outcome and improve outcome prediction.

**Methods:**

We conducted a prospective cohort study on comatose cardiac arrest patients on intensive care units. Resting-state fMRI three days after cardiac arrest was used to quantify whole-brain functional connectivity, global efficiency, clustering coefficient, and modularity. Neurological outcome at six months was classified as good or poor (Cerebral Performance Category 1–2 vs 3–5). Logistic regression models were used to examine between-group differences and study the additional value of graph-theoretical measures to clinical and EEG-based prediction.

**Results:**

In seventy included patients (good outcome n = 44, poor n = 26), whole-brain functional connectivity and clustering coefficient (but not global efficiency and modularity) were significantly lower in patients with poor outcome. Connectivity of nodes in posterior brain areas most prominently correlated with outcome. Clustering coefficient showed strong correlation with whole-brain functional connectivity. Patients with continuous EEG patterns differed in whole-brain functional connectivity levels from those with suppressed or epileptiform patterns. Combining functional connectivity or graph measures with clinical and EEG-based predictors slightly improved outcome prediction.

**Conclusion:**

fMRI-based whole-brain functional connectivity is a sensitive measure for encephalopathy severity after cardiac arrest, according to relations with established EEG categories and discrimination between good and poor outcome. Additional predictive values for outcome seem small. Graph measures do not provide complementary information.

## Highlights

• fMRI-based whole brain functional connectivity is related to EEG patterns in postanoxic coma.
• Whole brain functional connectivity discriminates between good and poor outcome, but additional prognostic value is small.
• Extraction of graph measures appears redundant.

## Graphical abstract

**Figure:**
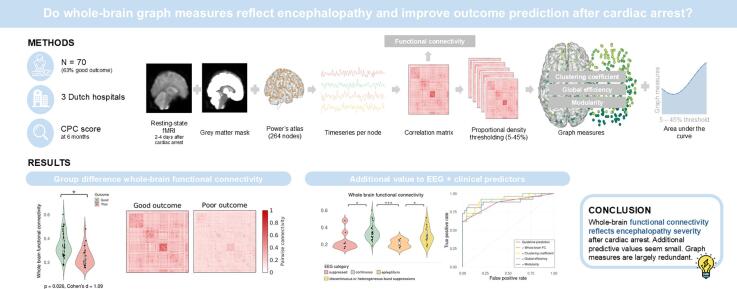

## 1 Introduction

Hypoxic-ischemic brain injury is the primary cause of mortality and long-term disability in comatose patients after cardiac arrest (Sandroni et al., 2020). After cardiopulmonary resuscitation, approximately one-third of patients show a return of spontaneous circulation (Yan et al., 2020). Subsequently, the rate of survival drops over time from 22 % at hospital admission to 8 % at one-year follow-up (Yan et al., 2020). Early assessment of brain injury and potential for neurological recovery is crucial for rational treatment decisions that may include withdrawal of life-sustaining therapy. However, accurate neurological outcome prediction remains a challenge. Current multimodal approaches based on the European guideline on post-resuscitation care leave up to 61 % of comatose patients with an indeterminate prognosis (Moseby-Knappe et al., 2020). This prognostic uncertainty highlights the need for new predictors to aid decision-making.

The current literature indicates a variety of alterations in overall brain functioning after cardiac arrest. These can be measured using various electrophysiological and imaging modalities capturing different aspects of brain activity. Electroencephalography (EEG) provides a measure of synaptic activity and has been shown useful for prognostication, allowing reliable prediction of good or poor outcome in about 50 % of all comatose patients after cardiac arrest (Rossetti et al., 2010, Ruijter et al., 2019, Sivaraju et al., 2015). Magnetic resonance imaging (MRI) shows distinct patterns in diffusivity measures (Hirsch et al., 2020, Keijzer et al., 2022a) and fMRI brain network integrity (Keijzer et al., 2022b, Koenig et al., 2014, Norton et al., 2012, Sair et al., 2018) when comparing patients with good and poor neurological outcome. The pathophysiology of cardiac arrest leads to widespread rather than localized hypoxic-ischemic brain injury, making whole-brain measures sensitive to changes in brain functioning. Hence, whole-brain measures of brain function hold potential to provide complementary information to improve prognostication in comatose patients after cardiac arrest.

Global brain functioning can be analysed from a network perspective using graph theory, where the brain is parcellated into nodes, representing distinct brain regions, and links as the pairwise connectivity between the nodes. This network can be analysed using various network metrics. Examples are communication efficiency across the brain (such as global efficiency) and the extent to which the brain forms more local communities (such as clustering coefficient) to assess network integration and segregation, respectively. For the brain to function effectively, it must demonstrate an optimal balance between integration and segregation within its networks (Stam, 2014). Patients with impaired consciousness show decreased integration and segregation in network features, which is on a whole-brain level (Achard et al., 2012, Malagurski et al., 2019, Martínez et al., 2020) as well as for specific brain networks (Crone et al., 2014, Peran et al., 2020). Large-scale brain network reorganization is proposed as the main factor distinguishing patients with varying degrees of recovery after traumatic brain injury (Oujamaa et al., 2023), acute brain damage (Martínez et al., 2020) or cardiac arrest (Achard et al., 2012). The clinical significance of integration and segregation alterations in distinguishing neurological outcomes after cardiac arrest remains unexplored and has not been related to other clinically established predictors before.

We hypothesize that whole-brain graph measures can differentiate between patients with favourable and unfavourable recovery and contribute to predicting neurological outcomes in comatose patients following cardiac arrest. We used data from a prospective cohort study involving early resting-state functional MRI scans taken three days post-cardiac arrest to test these hypotheses. We investigated whether whole-brain measures of integration and segregation discriminate between patients with good and poor recovery, and their ability to contribute to predicting neurological outcome. Additionally, we assessed the validity of fMRI-derived graph measures as a quantification of global brain functioning by analysing their relationship with global brain activity derived from early EEG measurements following cardiac arrest. Likewise, we assessed the (in)dependency of the measures as individual predictors. Our findings could serve as a basis for testing the whole-brain integration and segregation hypothesis in multimodal prediction models of neurological recovery following cardiac arrest.

## 2 Methods

### 2.1 Study design

We used data from a multicentre prospective cohort study designed to establish a multimodal prediction model of neurological outcome of comatose patients after cardiac arrest. The study is ongoing in three Dutch hospitals (Rijnstate Hospital, Radboud University Medical Centre and Maastricht University Medical Centre + ). For the current analysis, we used early resting-state fMRI data collected at three days after cardiac arrest and EEG collected at 24 h from patients included between June 2018 and July 2023. This study adheres to the Code of Ethics of the World Medical Association (Declaration of Helsinki) for experiments involving humans. The Committee on Research Involving Human Subjects region Arnhem-Nijmegen (number NL62151.091.17) approved the study protocol and is registered (clinicaltrials.gov identifier NCT03308305). A legal representative initially gave written informed consent, which was confirmed by the patient in case of recovery.

### 2.2 Study population

Patients were included within 72 h after cardiac arrest, during admission on the intensive care unit (ICU). The following inclusion criteria were used: age ≥ 18 years, Glasgow Coma Scale ≤ 8 at admission, and cardiac arrest of a cardiac origin or due to pulmonary embolism. Exclusion criteria were: life expectancy < 24 h, known progressive neurological disease (e.g., neurodegenerative disease or brain tumour), dependency in daily life prior to resuscitation, pregnancy, or a contraindication for MRI scanning (e.g., a pacemaker or neurostimulator).

### 2.3 Treatment

Treatment and monitoring followed local protocols that adhered to international guidelines for comatose patients following cardiac arrest (Berg et al., 2024). Promptly upon arrival at the ICU, targeted temperature management at 34 or 36 °C was initiated and maintained for 24 h (hospital dependent). Subsequent care comprised passive rewarming (0.25–0.5 °C per hour) and active normothermia maintenance. Sedation and analgesia predominantly included propofol, midazolam, morphine, sufentanil, or a combination of these.

Withdrawal of life-sustaining therapy was considered ≥ 72 h post-cardiac arrest, off sedation and during normothermia. Decisions were based on criteria from the European guidelines, i.e. bilateral absence of somatosensory evoked potentials, incomplete return of brainstem reflexes and treatment-resistant myoclonus (Nolan et al., 2021). Dutch guidelines include early EEG in multimodal outcome prediction since 2019 (Neurologie, 2019), which here was only considered 72 h after cardiac arrest. MRI results were not included in the prediction protocol.

### 2.4 Outcome

The primary outcome measure was the neurological outcome six months after cardiac arrest, quantified by the Cerebral Performance Category (CPC) score during a standardized telephone interview based on the EuroQol-6D questionnaire. Categorization consisted of good (CPC1-2, i.e. no/mild neurological impairment) and poor outcome (CPC3-5, i.e. severe neurological damage, vegetative state, coma or death).

### 2.5 MRI and EEG data acquisition

MRI scans were performed 3 ± 1 days after cardiac arrest on a 3 Tesla MRI scanner, either a Siemens Skyra (Radboudumc) or Philips Igenia (Rijnstate, Maastricht UMC + ). Resting-state fMRI consisted of a gradient-echo echo planar imaging sequence (Siemens: TE/TR 27/2280 ms, voxel size 3.2*3.2*3.0 mm, 220 volumes; Philips: TE/TR 27/2220 ms, voxel size 3.0*3.0*3.0 mm, 220 volumes). Anatomical scans consisted of 3D-T1 (Siemens: MPRAGE TR/TE 3.41/2400 ms, voxel size 0.9*0.9*1.0 mm; Philips Rijnstate: 3D TFE, TR/TE 3.8/8300 ms, voxel size 1.0*1.0*1.0 mm; Philips Maastricht UMC+: cs 3D ISO TR/TE 6.57/2.95 ms, voxel size 1.0*1.0*1.0 mm).

Continuous EEG recordings were initiated as soon as possible after the patient arrived in the ICU as part of standard care with a 10–20 system (Rijnstate, Maastricht UMC + ) or a reduced montage with ten electrodes (Radboudumc) (Tjepkema-Cloostermans et al., 2017). EEG initiation was always within 24 h after cardiac arrest, and continued to at least 72 h after cardiac arrest or until patient awakening.

### 2.6 MRI data analysis

MRI data quality was assessed visually for the T1 and resting-state fMRI scans using the individual reports of the MRI Quality Control tool (MRIqc) (Esteban et al., 2017). Specifically we evaluated framewise displacement as quantification of head motion. Preprocessing was conducted using FMRIPREP 22.1.1 (Esteban et al., 2022), including motion correction, resampling to standard space (MNI152Lin6cAsym), smoothing to 6 mm full-width half-maximum and non-aggressive denoising based on ICA-AROMA (Pruim et al., 2015). Subsequent nuisance regression included regressing out 24 motion derivates, cerebrospinal fluid, and white matter time series. The first 5 volumes were discarded to ensure a steady state and a bandpass filter of 0.007–0.1 Hz was applied. Patients were excluded if data quality was deemed inadequate after visual inspection of the final preprocessed data.

**Fig. 1:**
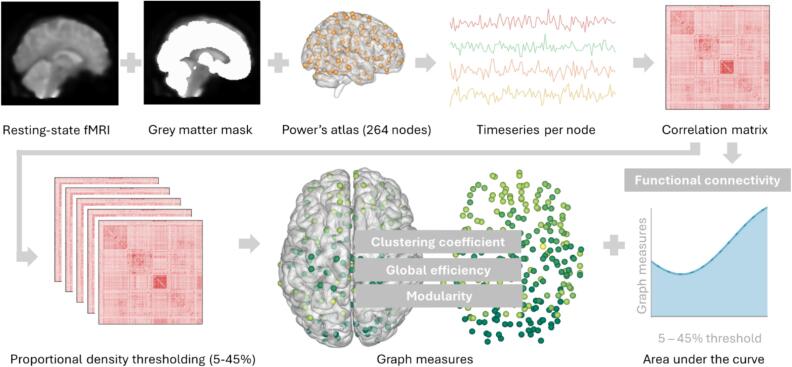
Graph analysis pipeline. Resting-state fMRI, grey matter mask, and Power’s atlas (i.e. 10 mm diameter spheres) were combined to extract time series per node. Correlation matrices were calculated by Pearson’s correlation coefficient for all combination of time series. Whole-brain functional connectivity was quantified as the average of all positive edges in the network. A range of density thresholds (5–45 %) was used in proportional density thresholding, followed by graph measures (clustering coefficient, global efficiency, modularity) estimation by the area under the curve over the density thresholds.

The consecutive data analysis involved dividing the data into 264 regions of interest using Power's atlas, which are 10 mm diameter spheres containing the cortex, subcortex, and cerebellum [29]. We used voxels within a grey matter mask derived from FMRIB's Automated Segmentation Tool (FAST) for extracting time series data [30]. We averaged and Z-scored the time series for each region of interest over all grey matter voxels. We then calculated the pairwise connectivity of the time series using Pearson's correlation coefficient for all combinations. The resulting 264x264 connectivity matrices were normalized using Fisher Z-transformation, and any negative correlations were adjusted to zero. Whole-brain functional connectivity was quantified as the average of all positive edges in the network. Proportional density thresholding was performed to reduce biased comparison between groups and to remove false connections via retaining the same amount of strongest edges for all patients (van den Heuvel et al., 2017). A range of density thresholds (5–45 %, step-size 5 %) was used. Graph measures were then extracted using the Brain Connectivity Toolbox. To eliminate subjective bias in selecting a threshold, the area under the curve for nine density thresholds was calculated as surrogate for the graph measure. Fig. 1 provides a visual summary of the analysis. Nodewise connectivity was quantified as the average overall correlation coefficients between a given node and the other nodes in the network.

Graph measures of interest were clustering coefficient (segregation) and global efficiency (integration). Clustering coefficient is calculated as the proportion of triangles around a node and its neighbours to quantify the tendency of nodes to form clusters (Rubinov & Sporns, 2009). Global efficiency (GE) is calculated as the average inverse shortest path length to quantify efficient information transmission (Latora & Marchiori, 2001). These network metrics have shown high correlations with each other (Li et al., 2011) and whole-brain functional connectivity (Soffer & Vázquez, 2005) and might thus be representing the same underlying topological feature. Therefore, we additionally assessed modularity as a measure of network segregation. Modularity is the extent to which the network is divided into groups of so-called modules. Modularity has shown less influence by the average degree or mean functional connectivity (Ferrarini et al., 2009, Newman, 2006). Comparing these commonly studied graph measures will provide information on whether they capture distinct or similar brain network characteristics.

### 2.7 EEG data analysis

Artifact-free 5-minute EEG epochs were selected by a computer algorithm at various time points (6, 12, 24, 36, 48 and 72 h) after cardiac arrest. Epochs were automatically bandpass filtered (0.5–35 Hz) and anonymously classified by two blinded reviewers. EEG recordings at 24 h after cardiac arrest were categorized into 5 categories as previously described: 1) suppressed; 2) suppressed background with pathological bursts or generalized periodic discharges (GPDs); 3) continuous activity; 4) epileptiform activity, consisting of seizures or GPDs on a continuous background; 5) discontinuous or heterogeneous burst suppressions (Ruijter et al., 2019a). Patients whose EEG showed epileptiform activity or a suppressed background with pathological bursts or GPDs at any timepoint were categorized accordingly. EEG data were classified as continuous activity if continuous activity was observed earlier than 24 h.

### 2.8 Statistical analysis

We calculated between-group differences using t-tests, Mann-Whitney U, or chi-square tests, where appropriate. We used Pearson’s correlation to test the relationship between whole-brain functional connectivity and graph measures.

Logistic regression models were used to examine between-group differences in graph measures while controlling for the following covariates: time between cardiac arrest and MRI scan, hospital, and comatose state (yes or no) during MRI. We employed Cohen’s d to quantify the effect sizes of the between-group differences.

We used Pearson’s correlation coefficient to calculate the correlation between nodewise connectivity and neurological outcome.

We used Kruskal Wallis tests to investigate the relationship of whole-brain graph measures with the EEG categories. In case the Kruskal Wallis test showed significant differences, subsequent pairwise Mann-Whitney U tests were performed to test for significant differences between the individual EEG categories.

To study the additional value of graph measures to clinical and EEG predictors based on the current criteria from the European guidelines, we compared logistic regression models of the guideline criteria with and without graph measures. In this ‘guideline prediction model’, we included bilateral fixed pupils and/or absent SSEPs and categorization of EEG into good (continuous pattern at t ≤ 12 h), poor (suppressed pattern at t ≥ 24 h) or indifferent (no established guideline characteristics). To quantify the predictive power of each model, we extracted the area under the curve (AUC) and sensitivity for good and poor outcome at specificity levels of at least 90 % (good outcome) and 100 % (poor outcome). The Likelihood Ratio Test was used to test whether the addition of graph measures improved the goodness of fit.

We used R (version 4.0.0) for all analyses. P-values below 0.05 were considered statistically significant. The false discovery rate (FDR) was used to correct for multiple comparisons.

## 3 Results

### 3.1 Subjects

Ninety patients were enrolled in the study at the time of analysis. Eleven were excluded because MRI could not be obtained 3 ± 1 days after cardiac arrest, five because of inadequate MR image quality, and four because follow-up data at six months were not available, leaving seventy patients for this analysis. Baseline characteristics are listed in Table 1.

**Table 1:** Baseline characteristics of patients with good and poor neurological outcome 6 months after cardiac arrest.

Characteristic	Good outcome (n = 44)	Poor outcome (n = 26)	*P-value*
Age (year)	59 (12)	66 (12)	0.02*
Male	36 (81 %)	17 (65 %)	0.21
Cardiac cause of arrest	44 (100 %)	23 (88 %)	0.09
Shockable first rhythm	43 (98 %)	18 (69 %)	< 0.001*
Time to ROSC (min)	14 [10–15]	22 [15–29]	< 0.001*
Time ROSC – MRI (hours)	59 (21)	66 (27)	0.02*
Comatose during MRI	16 (36 %)	25 (96 %)	< 0.001*
Treatment with sedatives during MRI (*propofol, midazolam, dexmedetomidine*)	16 (36 %)	22 (85 %)	<0.001*
Treatment with opiates during MRI (*morphine, sufentanil, remifentanil*)	7 (16 %)	7 (27 %)	0.45

Continuous variables are listed as mean (standard deviation) for normally distributed data or median [IQR]; otherwise, dichotomous variables are listed as n(%). Group differences are calculated using t-tests, Mann-Whitney U test or chi-square tests. Significant differences are marked by an asterisk (*). ROSC: the return of spontaneous circulation.

### 3.2 Whole-brain graph measures

**Fig. 2:**
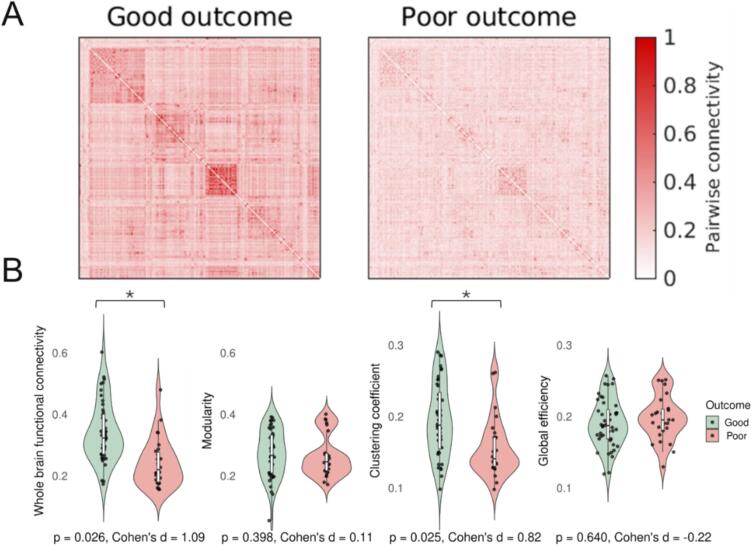
A) Average connectivity matrices of patients with good and poor neurological outcome, showing lower connectivity between pairs of nodes in patients with poor than in those with good neurological outcome. B) Violin plots of graph measures of patients with good and poor neurological outcome. P-values are corrected for multiple comparisons (FDR) and adjusted for time between cardiac arrest and MRI scan, hospital, and comatose state during MRI. * indicates statistical significance with FDR correction (p < 0.05).

Patients with poor neurological outcome showed on average a pattern with lower connectivity between pairs of nodes in their connectivity matrix compared to patients with good neurological outcome (Fig. 2A).

Whole-brain functional connectivity and clustering coefficient were significantly lower in patients with poor neurological outcome (*median (IQR)* 0.32 (0.13) vs 0.22 (0.10) and 0.19 (0.08) vs 0.14 (0.04), respectively; see Fig. 2). These differences remained statistically significant after correction for multiple comparisons. Effect sizes were large (whole-brain functional connectivity: *Cohen’s d* = 1.09; clustering coefficient*: Cohen’s d* = 0.82). Global efficiency and modularity did not significantly differ between patients with good and poor outcome (*median (IQR)* 0.19 (0.04) vs 0.20 (0.03) and 0.27 (0.12) vs 0.25 (0.06), respectively).

The clustering coefficient showed a positive correlation with whole-brain functional connectivity (*r* = 0.87*, p* < 0.001), whereas other graph measures showed no significant correlation. Global efficiency showed a positive correlation with the clustering coefficient (*r* = 0.43, *p* < 0.001) and a negative correlation with modularity (*r* = -0.34*, p* = 0.004).

### 3.3 Nodewise connectivity

**Fig. 3:**
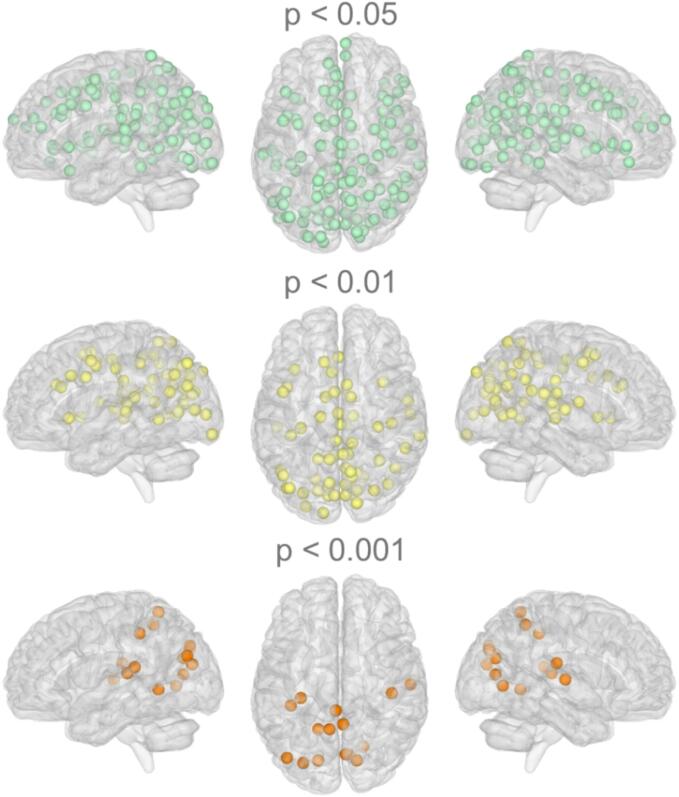
Nodewise connectivity correlated significantly with neurological outcome, presented at three uncorrected significance levels. In total 119 out of 264 nodes (Power’s atlas) significantly correlated with neurological outcome (p < 0.05). At p < 0.01, 58 nodes, and at p < 0.001, 15 nodes remain significant.

Significant correlations between nodewise connectivity and neurological outcome are shown in Fig. 3, presented at three uncorrected significance levels. Out of 264 nodes, 119 were significantly correlated to neurological outcome at p < 0.05, 58 at p < 0.01, and 19 at p < 0.001. Most significant correlations with nodewise connectivity were located in posterior, i.e. parieto-occipital, brain regions.

### 3.4 EEG and graph measures

**Fig. 4:**
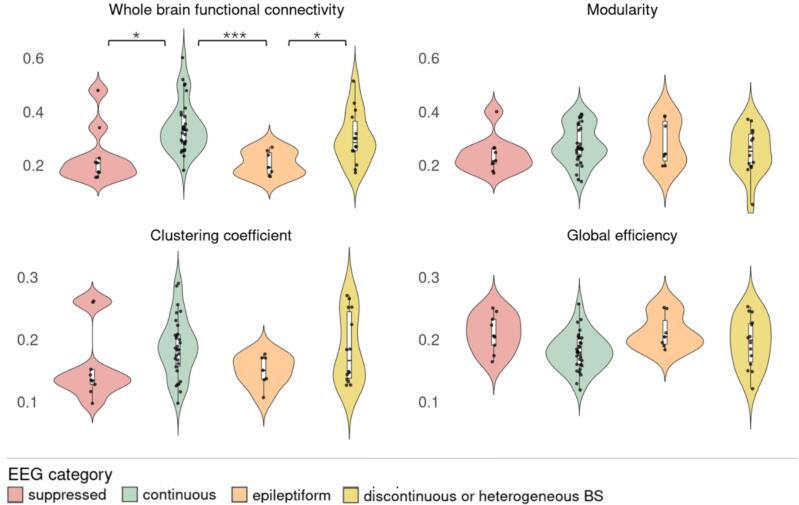
Graph measures per EEG-category. Groups presented here from left to right are: pattern of suppressed background with pathological bursts or GPDs (n = 9); continuous activity (n = 30); epileptiform pattern (n = 7); discontinuous or heterogenous burst suppressions (n = 14). GPDs = generalized periodic discharges, BS = burst suppression. Asterisks indicate statistical significance with FDR correction (* for p < 0.05, *** for p < 0.001).

Fig. 4 shows the distribution of graph measures in relation to EEG patterns at 24 h after cardiac arrest. Whole-brain functional connectivity significantly differed between EEG categories (*p* < 0.001*).* Specifically, those with continuous EEG activity significantly differed from suppressed (*p* = 0.014) and epileptiform (*p* < 0.001) EEG categories. Whole-brain functional connectivity was relatively low in patients with a suppressed background with pathological bursts or GPDs (*median (IQR)* 0.21 (0.05)) and in those with epileptiform patterns (*median* 0.19 (0.08)). Whole-brain functional connectivity was relatively high in patients with continuous activity (*median* 0.33 (0.11)) and discontinuous or heterogenous burst suppressions (*median* 0.29 (0.11)). Patients with epileptiform patterns significantly differed from those with discontinuous or heterogeneous burst suppression (*p =* 0.031). Global efficiency showed a small significant difference between EEG categories (*p = 0.04*), but no significant differences within specific EEG categories. The clustering coefficient and modularity did not show significant differences.

### 3.5 Predictive values of graph measures

Whole-brain functional connectivity and clustering coefficient were significantly related to neurological outcome six months after cardiac arrest as individual predictors (*p* < 0.001 and *p* = 0.004, respectively). The guideline prediction model has, compared to the individual graph measures, the highest AUC (0.87 vs 0.54–0.81) and sensitivity for poor outcome prediction (0.62 vs 0–0.15 at 100 % specificity).

**Fig. 5:**
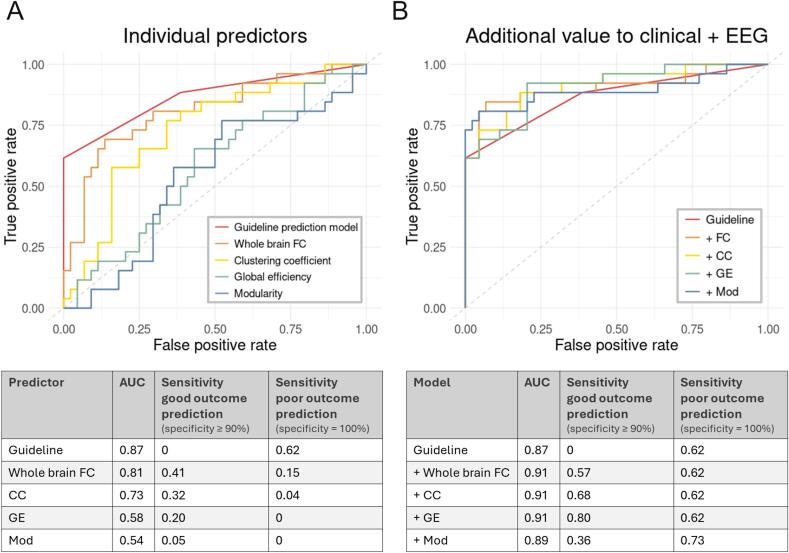
Receiver operating characteristic curves for generalized linear models of the guideline prediction model, i.e. established clinical and EEG characteristics, and four graph measures. ROC curves, AUC and sensitivities for good and poor outcome prediction are presented for A) the individual predictors and B) the additional value of the graph measures to the guideline prediction model. FC = functional connectivity, CC = clustering coefficient, GE = global efficiency, mod = modularity.

When adding the individual graph measures to the guideline prediction model, all models slightly improved in discrimination between patients with good and poor outcome (AUC 0.87 vs 0.89–0.91). Only the clustering coefficient and global efficiency significantly added predictive value to the guideline prediction model (*p =* 0.031 and *p* = 0.043). For clinical purposes, we are more interested in good and poor outcome prediction instead of overall discrimination. Good outcome prediction at a specificity level of at least 90 % improved in sensitivity (0 vs 0.36–0.80) when adding any of the four graph measures to the guideline prediction model. Poor outcome prediction, at a specificity level of 100 %, only improved in sensitivity when adding modularity (0.62 vs 0.73). Details are provided in Fig. 5.

## 4 Discussion

Our analyses revealed a significantly lower fMRI-based whole-brain functional connectivity at three days after cardiac arrest in comatose patients that had a poor neurological outcome as compared to those with good recovery. The clustering coefficient showed a similar difference, but was strongly correlated with whole-brain functional connectivity. Correlations with neurological outcome appeared predominantly in posteriorly located nodes in the brain. Whole-brain functional connectivity also related to EEG patterns at 24 h: it was lower in established EEG patterns indicative of poor than in those indicative of good outcome. Graph measures alone did not exceed guideline-based neurological outcome prediction, but showed potential to slightly improve predictive values when added to multimodal prediction models. This especially applied to prediction of good outcome.

This is, to our knowledge, the first graph-based fMRI analysis of comatose patients after cardiac arrest in relation to good and poor neurological outcome. Previous graph analyses addressed other disorders of consciousness (Crone et al., 2014, Peran et al., 2020) and mostly in comparison with healthy controls (Achard et al., 2012, Malagurski et al., 2019, Oujamaa et al., 2023, Peran et al., 2020). These previous graph analyses were largely in line with our results, showing relatively low functional connectivity in patients with disorders of consciousness (Crone et al., 2014, Oujamaa et al., 2023, Peran et al., 2020). Our results are also in line with resting state network based fMRI analyses, showing reduced functional connectivity across several resting-state networks in patients with poor neurological outcome after cardiac arrest (Keijzer et al., 2022a, Koenig et al., 2014, Norton et al., 2012, Sair et al., 2018). In addition to relations with good or poor outcome, we showed a relation between whole-brain functional connectivity and EEG patterns indicative of good or poor outcome. This confirms the physiological validity of our used functional connectivity measures.

The strong correlation between the clustering coefficient and overall whole-brain functional connectivity generally aligns with previous research, showing high correlations between graph based network metrics and overall functional connectivity (Li et al., 2011, Martin Hernandez and Van Mieghem, 2011, Soffer and Vázquez, 2005). This indicates that the observed results for the clustering coefficient are probably driven by an underlying difference in whole-brain functional connectivity. Given the dominance of whole-brain functional connectivity differences in our findings, we hypothesize that this is likely the most important network property for predicting the outcome of postanoxic coma. Specific graph measures are likely redundant for prognostication. This redundancy is supported by existing literature, which shows inconsistent findings regarding the specific graph measures and brain regions affected (Achard et al., 2012, Crone et al., 2014, Malagurski et al., 2019, Martínez et al., 2020, Peran et al., 2020). Overall, these alterations appear to be widespread, impacting both the integration and segregation of the brain network. Therefore we believe that it is more effective to use broad measures like whole-brain functional connectivity as potential clinical biomarkers, rather than focusing on derived metrics of integration and segregation. This rationale is further strengthened by the fact that functional connectivity, as a direct measure, reduces the subjective decisions and methodological inferences required in fMRI analyses, making it more reliable than higher-level metrics.

Graph measures alone did not outperform the guideline prediction model. Incorporating individual graph measures alongside clinical and EEG predictors enhanced the prediction of good outcome. The low sensitivity for good outcome prediction in the guideline model is likely due to the focus on markers for poor outcome prediction in the guideline. For poor outcome prediction, modularity was the only factor that increased sensitivity. The improvement in mainly good outcome prediction is in line with our previous work, which indicated that fMRI seemed particularly suitable for good outcome prediction (Keijzer et al., 2022a).

We observed that diffusely throughout the brain, but most significant in posterior regions, nodes were correlated with neurological outcome. This is in line with previous studies showing most structural (Keijzer et al., 2022a, Luigetti et al., 2012, Mlynash et al., 2010) and functional (Achard et al., 2012, Crossley et al., 2014, Keijzer et al., 2022a, Koenig et al., 2014, Sporns, 2014) brain injury after cardiac arrest in posterior brain regions. It suggests that posterior brain regions are especially vulnerable to hypoxia or ischemia. Over the past years, alterations in hubs, i.e. highly connected brain regions, have been increasingly studied as substrates for brain disorders (Crossley et al., 2014, Hagmann et al., 2008a, Hagmann et al., 2008b, Sporns, 2014). The vulnerability and metabolic cost of such highly connected areas have been observed across various conditions (Crossley et al., 2014) and may likewise be sensitive to disruption from hypoxia-induced metabolic distress after cardiac arrest (Achard et al., 2012, Keijzer et al., 2022a, Luigetti et al., 2012). From a pathophysiological perspective, studying network vulnerability at a finer-grained level offers promising insights. Previous studies suggest that local and global network properties may be differentially affected under pathological conditions, highlighting the complexity of brain network responses to injury (Achard et al., 2012). However, the additional value and clinical relevance of spatially differentiated analyses are uncertain.

Another explanation is articulated in the “posterior hot zone theory” of consciousness, which posits that the neural substrate of consciousness resides in the posterior parietal cortex, praecuneus and parts of the temporal and occipital lobe (Koch et al., 2016, Seth and Bayne, 2022). Reduced connectivity in these areas has been associated with reduced states of consciousness (Hagmann et al., 2008a, Hagmann et al., 2008b, Ihalainen et al., 2021, Vanhaudenhuyse et al., 2010, Wu et al., 2015) and could thus particularly discriminate between comatose patients with good and poor recovery. In terms of consciousness, sedation levels significantly differed between the outcome groups and thus might have influenced our findings. However, comatose patients with good outcome often require more sedation than those with poor outcome. Therefore, we opted for not making adjustments.

The strengths of this study include its prospective design and its novelty as the first study on outcome prediction using fMRI-based graph measures in comatose patients after cardiac arrest. In addition, we assessed the validity of fMRI-derived graph metrics by relating them to established EEG patterns. Our study also has limitations. First, as in all graph analyses, choices in preprocessing and thresholding are partly subjective and do have a substantial influence on the final graph measures (Gargouri et al., 2018). Head motion can impact functional connectivity measures (Power et al., 2011, Satterthwaite et al., 2012) and cannot be fully eliminated. However, this study was designed to minimize its effects through robust quality control, preprocessing, and statistical analyses. Second, the multicentre design with slight differences in MRI characteristics might have influenced our findings. However, protocols were harmonized, preprocessing ensured analyses were done in one standard space and we corrected for the study site to minimize this effect. Third, studies on outcome prediction after cardiac arrest generally cannot completely exclude the influence of self-fulfilling prophecy. However, withdrawal of life-sustaining treatment was never being informed by brain MRI. Nevertheless, factors considered in this decision such as EEG background and clinical severity, are related to MRI findings. Fourth, we did not include a healthy control group, as our primary goal was to identify nuanced differences within the patient cohort. Consequently, we are unable to directly compare our findings to previous studies that examined differences between patients and healthy controls.

## 5 Conclusion

fMRI-based whole-brain functional connectivity is a sensitive measure for encephalopathy severity after cardiac arrest, according to clear relations with established EEG categories. Whole brain functional connectivity discriminates between patients with good and poor outcome, but the additional value for outcome prediction seems marginal. Extraction of specific graph measures beyond whole-brain functional connectivity appears redundant.

## Funding

This study was funded by the Rijnstate-Radboud promotion fund, provided by the Rijnstate hospital, Arnhem, and the Radboudumc, Nijmegen.

## CRediT authorship contribution statement

**Puck Lange:** Writing – original draft, Visualization, Software, Methodology, Investigation, Formal analysis, Data curation, Conceptualization. **Marlous Verhulst:** Writing – review & editing, Software, Methodology, Investigation, Formal analysis, Data curation, Conceptualization. **Anil Man Tuladhar:** Writing – review & editing, Validation, Software, Methodology, Conceptualization. **Prejaas Tewarie:** Writing – review & editing, Validation, Software, Methodology. **Hanneke Keijzer:** Writing – review & editing, Project administration, Funding acquisition, Data curation, Conceptualization. **Catharina J.M. Klijn:** Writing – review & editing, Resources, Conceptualization. **Cornelia Hoedemaekers:** Writing – review & editing, Resources, Conceptualization. **Michiel Blans:** Writing – review & editing, Resources, Investigation. **Bart Tonino:** Writing – review & editing, Investigation, Data curation. **Frederick J.A. Meijer:** Writing – review & editing, Investigation, Data curation. **Rick C. Helmich:** Writing – review & editing, Validation, Supervision, Methodology, Conceptualization. **Jeannette Hofmeijer:** Writing – review & editing, Validation, Supervision, Methodology, Conceptualization.

## Data Availability

Data will be made available on request.
